# Pulmonary metastasis of a papillary thyroid carcinoma and primary lung adenocarcinoma: two coincident carcinomas at the same location

**DOI:** 10.1186/1746-1596-8-26

**Published:** 2013-02-16

**Authors:** Liyan Xue, Zhonghua Luan, Ying Liu, Shuangmei Zou, Jun Jiang, Ning Wu, Ning Lu, Dongmei Lin

**Affiliations:** 1 Department of Pathology, Cancer Institute (Hospital), Peking Union Medical College, Chinese Academy of Medical Sciences, Beijing, China; 2 Department of Pathology, Yuncheng Central Hospital, Yuncheng, Shanxi Province, China; 3 Department of Nuclear Medicine, Cancer Institute (Hospital), Peking Union Medical College, Chinese Academy of Medical Sciences, Beijing, China; 4 Department of Imaging Diagnosis, Cancer Institute (Hospital), Peking Union Medical College, Chinese Academy of Medical Sciences, Beijing, China

**Keywords:** Tumor-to-tumor metastasis, Papillary thyroid carcinoma, Lung adenocarcinoma, Napsin-A, Thyroglobulin

## Abstract

Tumor-to-tumor metastasis is a fairly rare phenomenon. The lung cancers are the most common donors, but are exceedingly rare as recipients. Here we report a case of a lung adenocarcinoma acting as the recipient of papillary thyroid carcinoma, with multiple spreading foci of the two cancers in the lung simultaneously. The morphology and immunohistochemisty (Napsin-A, Thyroglobulin) are very important in differential diagnosis of lung primary adenocarcinoma and metastatic papillary thyroid carcinoma.

**Virtual Slides:**

The virtual slide(s) for this article can be found here: http://www.diagnosticpathology.diagnomx.eu/vs/2069496615891134

## Background

Coexistence of two or more primary neoplasms in one patient is not uncommon, however, tumor-to-tumor metastasis is a fairly uncommon but interesting phenomenon. The most frequent recipient is renal clear cell carcinoma, followed by sarcoma, meningioma, and thyroid neoplasm, while the most common donor is lung carcinoma, followed by carcinoma of the breast, gastrointestinal tract, prostate, and thyroid [1,2]. The lung cancers are the most common donors, but are exceedingly rare as recipients. To date, only 3 cases of lung cancer acting as the recipient of papillary thyroid carcinoma were reported in English-language literature. They were invasive adenocarcinoma, squamous cell carcinoma, and non-mucinous adenocarcinoma *in situ*, respectively [3-5]. Here we report a case that papillary thyroid carcinoma metastasized into a lung primary invasive adenocarcinoma, with multiple spreading foci of the two cancers in the lung simultaneously.

## Case presentation

A 65-year-old Chinese man underwent total thyroidectomy and bilateral neck dissection with ^131^I treatment after that for papillary thryoid cancer in a local hospital. The papillary thryoid cancer extended to perithyroid soft tissues (pT3) with bilateral neck lymph node metastasis (pN1b). Chest Computed Tomography (CT) scan was not performed that time. Seven months later, a mass in his lung was found without any symptoms in physical examination.

## Immunohistochemistry

Specimens were fixed in 10% buffered formalin, embedded in paraffin and stained with HE method routinely for histological examination. Immunohistochemical staining was performed using an automated immunostainer (Ventana Benchmark XT; Ventana Medical Systems, Inc, Tucson, AZ). Antibodies against Cytokeratin 19 (CK19), Cytokeratin 7 (CK7), Napsin-A, Thyroglobulin and Thyroid Transcription Factor-1 working solution (CK19 and CK7: Zhong Shan –Golden Bridge Biological Technology Co. Ltd., Beijing, China; Napsin-A and Thyroid Transcription Factor-1: Maxim Biological Techology Co. Ltd., Fuzhou, China; Thyroglobulin: Dako Corp, Carpinteria, CA, USA). Antigen recovery was conducted using moderate heat retrieval according to manufacturer’s recommendation. Slides were incubated with the primary antibody for 30 mininutes at room temperature.

## Results

**Figure 1:**
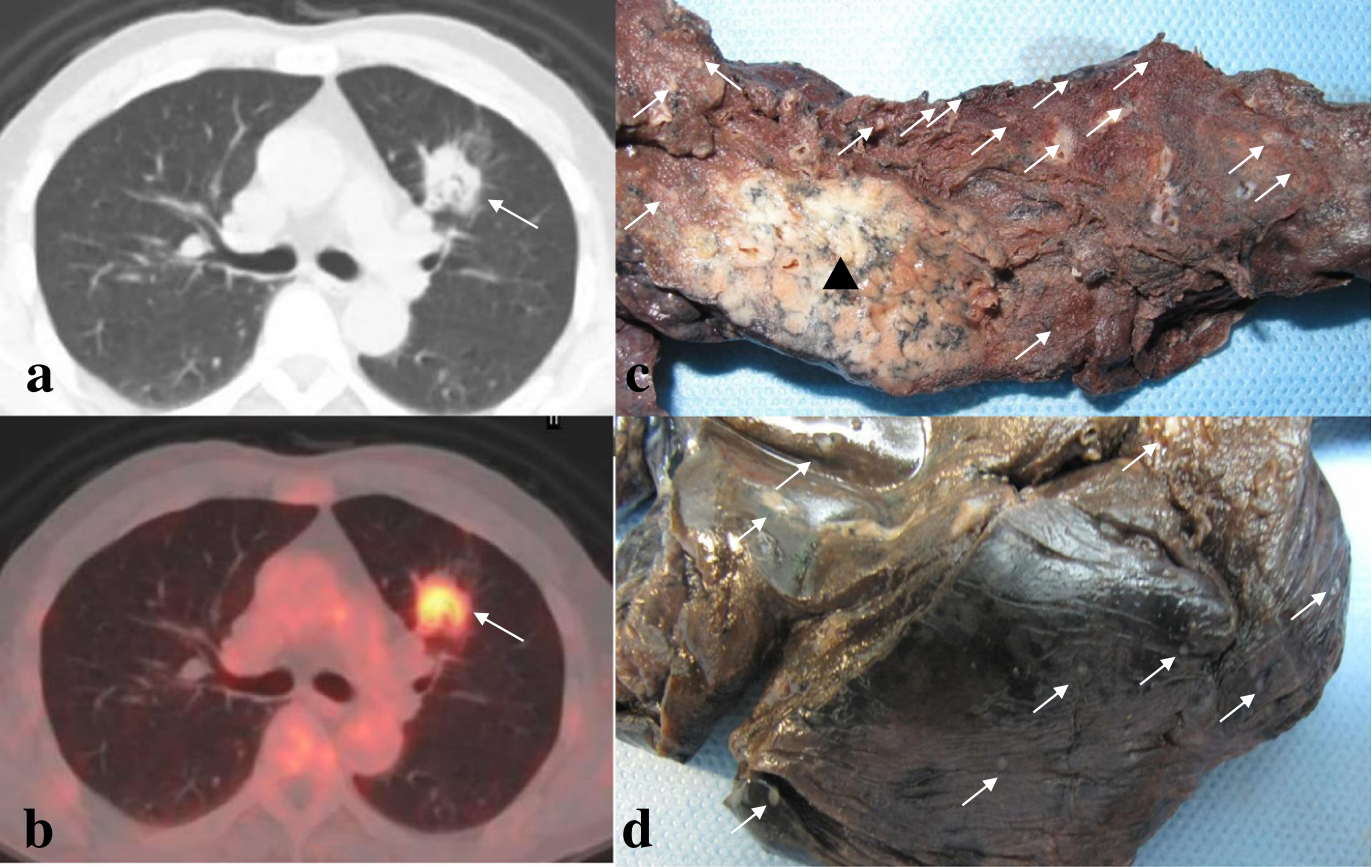
**PET-CT imaging features and macroscopic features. **CT revealed a large lobular mass in the superior lobe of left lung. The largest dimension of the mass is 3.2 cm. It invaded the anterior segment and superior segment of the left lung, and invaded pleura mediastinalis. The imaging of the rest lung looks normal (**a**). PET and CT fusion image showed the nodule had intense FDG uptake. The SUVmaxs was 5.2, which suggested a malignant tumor on PET-CT, and probably lung primary cancer (**b**). Gross examination showed a tumor (▲) in the peripheral of the lung, with the largest diameter of 3.5 cm. The cut section surface of the tumor was hard, grey and fine granular, and there were many small grey nodes (arrows) in the rest lung, with a diameter of 0.1 cm to 0.3 cm (**c**). There were plenty of small nodes (arrows) on the visceral pleura (**d**).

PET-CT and CT revealed a large lobular mass in the superior lobe of left lung. The largest dimension of the mass is 3.2 cm. It invaded the anterior segment and superior segment of the left lung, and invaded pleura mediastinalis. The imaging of the rest lung looks normal (Figure 1). Many lymph nodes can be seen in the 2R, 4R, 4 L, 5 and 6 regions of mediastina, with the maximum dimension is 1.4 cm. According to the recommendation of PET-CT and CT examination, the patient underwent lobectomy. The patient was alive half a year later.

Gross examination showed a tumor in the periphery of the lung, measuring 3.5 × 3.5 × 2.5 cm. The cut section surface of the tumor was hard, grey and fine granular. There were many small grey nodes in the rest lung and pleura, with a diameter of 0.1 cm to 0.3 cm. There were plenty of small nodes on the visceral pleura (Figure 1).

**Figure 2:**
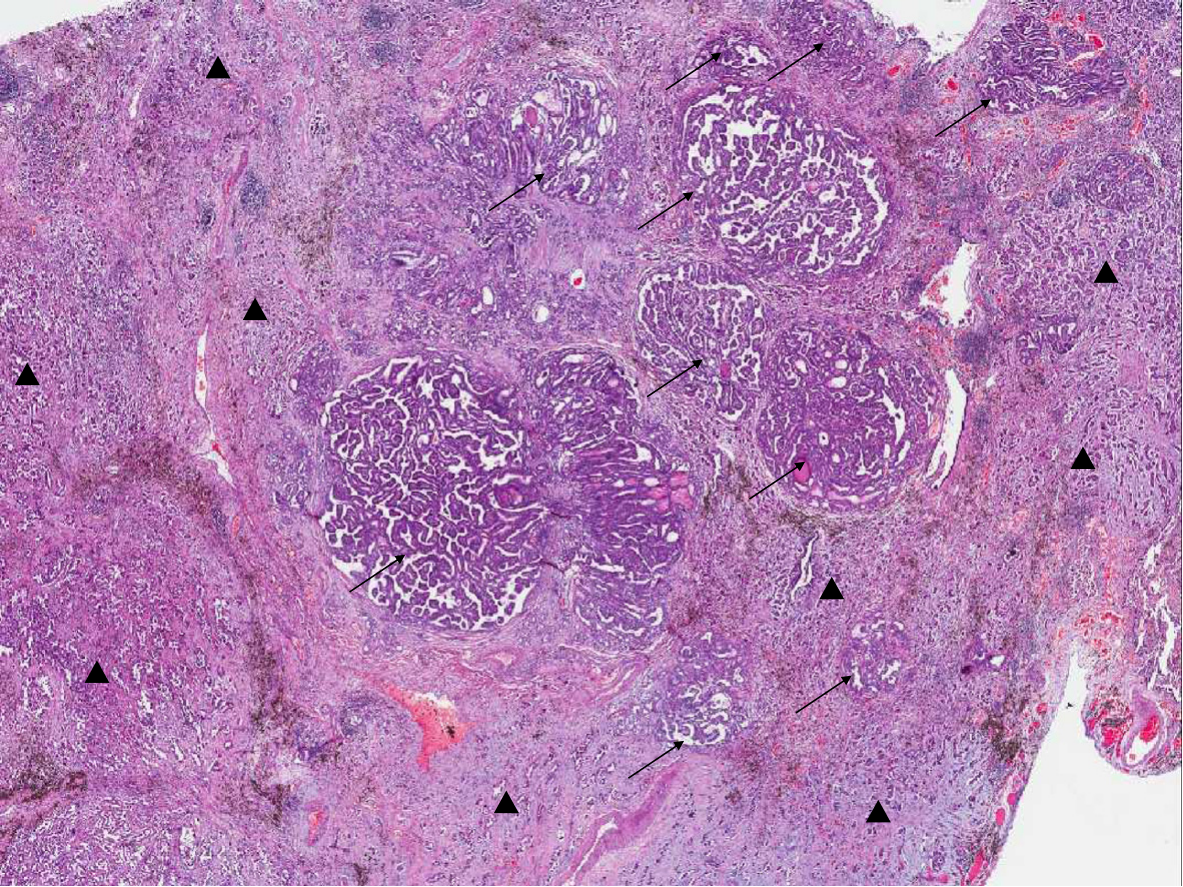
**Microscopic features of the metastatic papillary thyroid carcinoma and primary lung adenocarcinoma. **Metastatic papillary thyroid carcinoma (→) and primary lung adenocarcinoma (▲) were mixed with each other.

**Figure 3:**
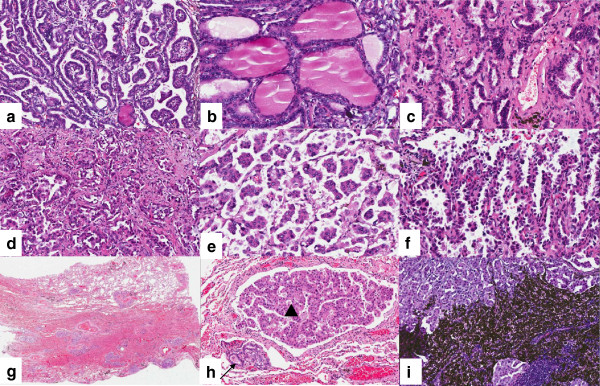
**Microscopic features of the metastatic papillary thyroid carcinoma and primary lung adenocarcinoma. **The typical papillary pattern, ground glass nuclei, groove and colloid were found in metastatic papillary thyroid carcinoma (**a, b**). The tumor was mainly consisted of acinar pattern in primary lung adenocarcinoma. Micro-papillary and leptic (bronchioloalveolar carcinoma) patterns can also be seen (**c, d, e, f**). In the other area of the lung, the two tumor components, metastatic papillary thyroid carcinoma (→) and lung adenocarcinoma (▲), spread simultaneously. They were mixed with each other (**g, h**). Metastatic carcinoma in the lymph nodes was lung adenocarcinoma (**i**).

**Figure 4:**
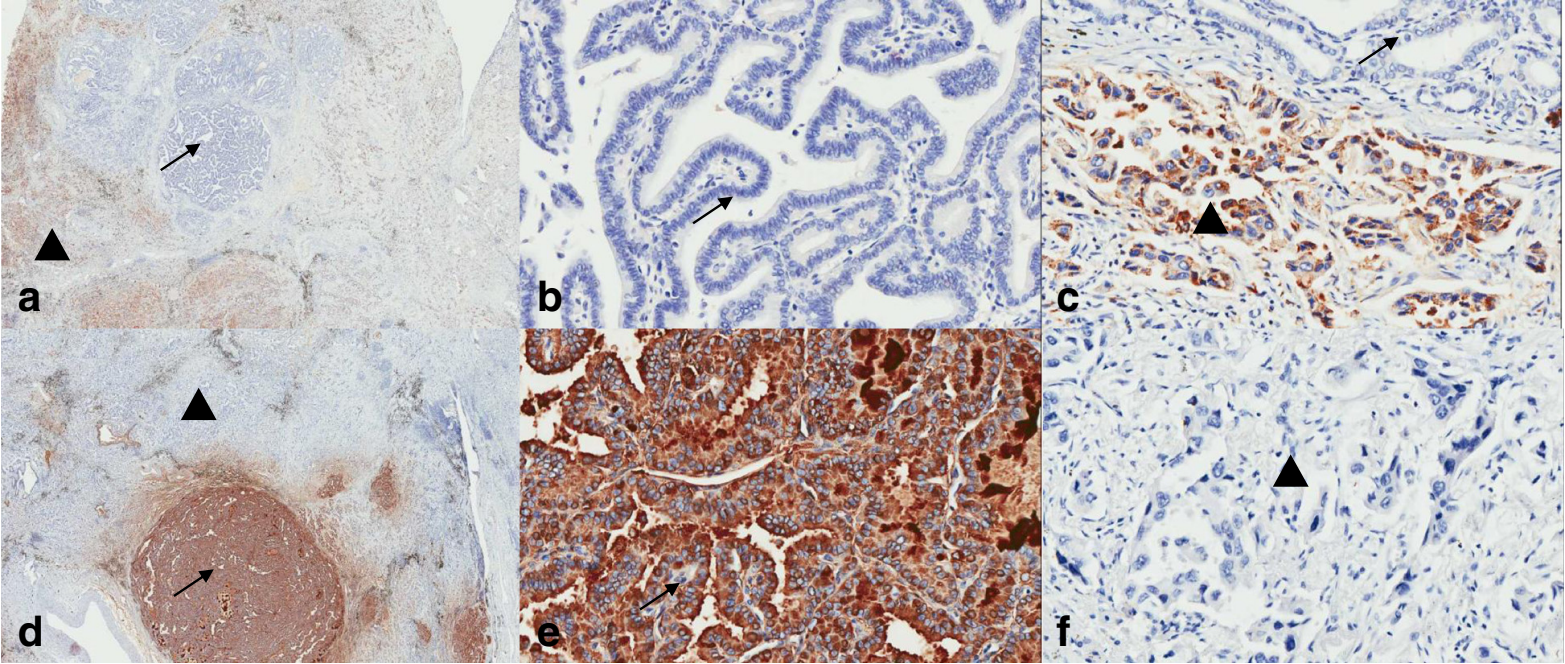
**Immunohistochemical features of the metastatic papillary thyroid carcinoma and primary lung adenocarcinoma. **Napsin A was positive in primary lung adenocarcinoma (▲), but negative in metastatic papillary thyroid carcinoma (→) (**a, b** and **c**). Thyroglobulin was positive in metastatic papillary thyroid carcinoma (→), but negative in primary lung adenocarcinoma (▲) (**d, e** and **f**).

Microscopically, two different cell populations were identified. They were mixed with each other. One was with typical papillary thyroid carcinoma morphology with typical papillary pattern, ground glass nuclei, groove and colloid. The other was with primary lung adenocarcinoma morphology, mainly consisted of acinar pattern. Micro-papillary and leptic (bronchioloalveolar carcinoma) patterns can also be seen. Cytoplasm was abundant and acidophilia. Nuclei were dark stained, with small nucleoli and evenly distributed chromatin. In the surrounding lung, the two tumor components, metastatic papillary thyroid carcinoma and lung adenocarcinoma, spread as small foci simultaneously. They were also mixed with each other. The spreading lung adenocarcinoma was mainly acinar pattern and micro-papillary pattern but not leptic pattern or adenoid atypical hyperplasia (AAH) (Figures 2 and 3). Metastatic carcinoma in the lymph nodes was lung adenocarcinoma. CK7, CK19 and Thyroid Transcription Factor-1 were positive in both cell populations. Napsin A was positive in primary lung adenocarcinoma, but negative in metastatic papillary thyroid carcinoma. Thyroglobulin was positive in metastatic papillary thyroid carcinoma, but negative in primary lung adenocarcinoma (Figure 4).

## Discussion

Very few reports of tumor-to-tumor metastasis are present in the English literature. The terms “tumor-to-tumor metastasis” and “collision tumor” have often been confused with one another, and used incorrectly in the literature. Collision tumor is defined as two neighboring neoplasms that invade one another. Tumor-to-tumor metastasis has proved more difficult to be defined, with several proposed similar criteria in order to be classified correctly. The criteria of Campbell et al. to define tumor-to-tumor metastasis are as follows [2]. (1) more than one primary tumor must exist; (2) the recipient tumor is a true benign or malignant neoplasm; (3) the metastatic neoplasm is a true metastasis with established growth in the host tumor, not the result of contiguous growth (collision tumor) or embolization of tumor cells; (4) involvement by metastatic tumors of lymph nodes already involved by lymphoproliferative disease (lymphoreticular malignant tumors) is excluded. According to the literature, renal clear cell carcinoma is the most common tumor recipient of metastasis while lung carcinoma is the most common donor tumor [1,2,6]. Tumor-to-tumor metastasis is not as infrequent as previously thought and may be present in up to 15 percent of patients with simultaneous renal clear cell carcinoma and second neoplastic disease [6]. Commonly, the donor tumor is an aggressive malignant tumor. On the other hand, the recipient tumor is usually a low-grade malignancy or benign neoplasm. It is extremely rare that an aggressive malignant tumor as the recipient tumor while a low-grade malignancy as the donor tumor. Only three cases of papillary thyroid carcinoma, a low-grade malignancy, metastasizing to a lung cancer, an aggressive malignant tumor, were reported [3-5]. There were more reports about lung cancer metastasizing to a thyroid tumor [7-10].

The tumor cells (forming the ‘seed’) are hematogenously disseminated in a widespread manner to different anatomic sites, but attain successful growth and propagation only under the favorable local microenvironments of specific organs or the recipient tumors (the ‘soil’). Renal clear cell carcinoma is the most common recipient tumor in tumor-to-tumor metastasis. This might be attributable to its increased vascularity. Biochemical properties of renal cell carcinoma, such as increased cytoplasmic lipid and glycogen of constituent cells, has also been proposed as contributing to this tumor being a favorable site for metastatic growth [11]. The lung is one of the most frequent sites of metastasis for the extrathoracic cancers. However, lung carcinoma is one of the rarest recipients for tumor-to-tumor metastasis. Presence of different vascular structures between normal lung and lung cancer may be the possible reason for the different potential as a site of metastasis. Unlike normal lung, lung cancer doesn’t contain a rich network of small thin-walled blood vessels. The other possible explanation which we considered was that lung cancer is rapidly growing tumor therefore it cannot provide a suitable environment for the acceptance of metastasis. Regarding the reason for the relatively high incidence of lung cancer as a donor in tumor-to-tumor metastasis, the rich vascularity of lung carcinoma in association with the probable shedding of tumor cells during respiratory movement might increase the frequency of invasion into the pulmonary vein and subsequently into the general circulation. The tumor emboli from lung cancer have a better chance to reach all the body organs through the left-sided cardiac output than cancers of the other organs.

It is still unexplained whether tumor-to-tumor metastasis is merely of a chance occurrence or is a selective growth within the recipient tumor. In our case, a lung mass with metastatic papillary thyroid carcinoma was the first metastatic focus discovered during the post-operative follow-up of this patient. Our case showed that the metastatic papillary thyroid adenocarcinoma foci were not limited to the primary lung adenocarcinoma and multiple microscopic foci of thyroid papillary foci were observed in the surrounding normal lung tissue. Multi foci of metastases can also be seen both within the lung cancer and in the background lung tissue in the 3 reported cases of a lung cancer acting as the recipient of a donor of papillary thyroid carcinoma [3-5]. In addition, multiple spreading small lung adenocarcinoma can also be seen in the background lung tissue in our present case while the 3 reported cases didn’t show this. Papillary thyroid carcinoma is a slowly growing tumor. Disease-specific survival with distant metastases at 5 and 10 years was reported to be 65 and 45%, respectively [12]. The metastatic foci of papillary thyroid carcinoma had probably already been there before lung primary adenocarcinoma occurred, and the metastases to lung cancer had occurred by chance.

The presence of a lung mass in a patient with a prior cancer can represent a clinically and/or pathologically diagnostic challenge. Some of these lung masses represent primary processes rather than metastatic deposits. The diagnosis of metastatic carcinoma to the lung by core biopsy, frozen section, and permanent sections can be easily misinterpreted as a primary tumor. The possibility of metastatic papillary thyroid carcinoma should always be considered when two different cell populations are identified, and specifically, one should be aware of in the presence of papillary component in a lung carcinoma. The diagnosis of metastatic papillary thyroid carcinoma mainly depends on the morphology of the nucleus and immunohistochemical expression. The papillary thyroid carcinoma is composed of cells with ground glass nuclei in the cells and colloid in the thyroid follicles, which are the important characteristics different from other carcinomas. Lepdic or adenocarcinoma *in situ* components in the surgical specimens can also help to differentiate primary lung adenocarinoma from other carcinomas. Thyroid Transcription Factor-1 is one of the immunohistochemical markers most commonly used to assist in the differential diagnosis of carcinomas of the lung and thyroid from other carcinomas. But it cannot differentiate between lung carcinoma and thyroid carcinoma. CK19, galectin-3 (Gal-3) and Hector Battifora mesothelial-1 (HBME-1) have been studied to distinguish between malignant and benign lesions of the thyroid gland. The diagnostic efficiency of CK19 for papillary thyroid carcinoma was slightly better than that of Gal-3 and HBME-1. The utilization of these markers combined with morphologic evaluation may be helpful in the differential diagnosis of papillary thyroid carcinoma and benign lesions [13,14]. But they cannot distinguish metastatic papillary thyroid carcinoma from lung adenocarcinoma. Thyroglobulin is an antibody specially expressed in normal thyroid and thyroid carcinoma, which we can use in the differential diagnosis of lung carcinoma and thyroid carcinoma. Napsin A is an aspartic proteinase involved in the maturation of surfactant protein B. It is detected in the cytoplasm of type 2 pneumocytes and alveolar macrophages and is a putative marker for lung adenocarcinomas. It can also help to differentiate lung adenocarcinoma from metastatic papillary thyroid carcinoma [15]. Recently, microarray based gene expression profiling has found increasing use, particularly in the most difficult cases. Gene expression profiling provides correct primary site identification in a higher percentage, of metastatic cases than immnunohistochemistry-guided methods (90% *vs.* 64%), and uses less tissue [16]. In our present case, the morphology and immunohistochemical expression of the two components were typical. It was a rare case, but not difficult for pathologist, so gene expression profiling is not necessary.

## Conclusion

In summary, when there is a tumor in the lung of a patient with papillary thyroid carcinoma history, tumor-to-tumor metastasis should be considered. Such a tumor-to-tumor metastasis should be differentiated from primary mixed adenocarcinoma, which is composed of papillary adenocarcinoma and other subtypes of adenocarcinoma of lung. Tumor-to-tumor metastasis may become more frequent because of the improving prognosis and survival of patients with malignancies. Awareness about this phenomenon of tumor-to-tumor metastasis, careful examination of morphology and proper immunohistochemical staining such as Thyroglobulin and Napsin A are important for correct diagnosis and appropriate therapy.

## Consent

Written informed consent was obtained from the patient for publication of this case report and accompanying images. A copy of the written consent is available for review by the Editor-in-Chief of this journal.

## Competing interests

The authors declare that they have no competing interests.

## Authors’ contributions

LX made contributions to acquisition of clinical data, pathological analysis and manuscript writing. ZL drafted the manuscript. YL prepared the PET-CT images. SZ made contributions to pathological analysis. JJ prepared the CT images. NW made contributions to PET-CT and CT imaging analysis. NL and DL conceived of the study, and participated in its design and coordination and helped to draft and edit the manuscript. All authors read and approved the final manuscript.
